# The use of the laser confocal scanning microscopy to measure resin remnants on customized lingual bracket

**DOI:** 10.1186/s12903-020-01132-4

**Published:** 2020-05-14

**Authors:** Can Kuskonmaz, Alberto De Stefani, Gilberto Artioli, Matteo Zanarini, Giulio Alessandri Bonetti, Giovanni Bruno, Antonio Gracco

**Affiliations:** 1 Faculty of Dentistry, University of Padova, via Giustiniani 2 –, 35100 Padova, Italy; 2 Department of Geoscience, University of Padova, Padova, Italy; 3 Department of Orthodontics, University of Bologna, Bologna, Italy

**Keywords:** Lingual orthodontics, debonding, ARI, confocal microscope

## Abstract

**Background:**

The study aimed to evaluate the permanence of resin and enamel remains on lingual brackets at the end of orthodontic treatment and after the debonding procedure. The evaluation of resin remnants on customized lingual brackets bases has never done before in other studies because they are curved, and traditional techniques are not applicable.

**Methods:**

The sample consisted of 100 lingual brackets (25 incisors, 25 canines, 25 premolars, 25 molars) scanned with a confocal laser microscope (OLS4000). We measured the brackets' surface and the area of resin remnants with the software of the microscope. Median and quartiles were presented to describe the data. ARI calculation was indirect for each tooth, measuring the resin remnants to the total surface of the bracket. The Kruskal-Wallis test and Fisher test were applied respectively to compare the percentages of remnants and the frequencies of the ARI between the four groups.

**Results:**

After the analyses, 13 brackets had no adhesive remnants (ARI 0), 29 brackets had less than 50% of resin remnants (ARI 1), 50 brackets had more than 50% of resin remnants (ARI 2), and 8 brackets had 100% of adhesive (ARI 3). Canines brackets presented the lower amount of resin followed by premolars, incisors, and molars.

**Conclusion:**

Lingual brackets showed a high frequency of ARI = 2. The median percentage of the bracket surface covered by resin was 41%. We observed a slight tendency of more resin remnants on molar brackets, due to half-pad configuration. The authors suggest paying attention during the debonding procedure of molar brackets since a stronger connection between the adhesive and the bracket mesh means a higher risk of enamel damage.

## Background

At the end of the orthodontic treatment with labial or lingual appliances, orthodontists have to remove brackets, residual composite, and adhesive remnants from the teeth surfaces, trying to restore the pretreatment condition without damaging the enamel surface [1–3]. There are different factors associated with this procedure, for example, the kind of bonding protocols and materials but also the tools for bracket removal and burs sequence [4, 5].

In medical literature, there are not universally approved protocols for the clean-up of adhesive resin after bracket removal, and no instrument can achieve complete resin removal without scratching or grooving the enamel surface. Damage to the enamel surface during debonding procedure has been reported in vitro [6–8] and in vivo studies [9, 10]. Bond failure during the bracket removal procedure can occur in different modalities: adhesive failure is when it occurs at the adhesive-bracket interface or the adhesive-enamel interface, cohesive failure is when it occurs within the adhesive. The most frequent scenario is generally a combination is a mixed failure when a combination of adhesive and cohesive failure happens [11]. Different studies evaluated the damage of the dental surface during debonding, demonstrating that a higher risk occurs with an adhesive failure between the resin and the enamel [11–13]. This event can occur with metal brackets but more often with ceramic ones [14].

At the date, only a few studies evaluated the debonding forces, and shear bond strength and bracket base remnants of customized lingual appliance [15]. The principal limitations of these studies are the in-vitro protocols and that authors generally perform the tests only on premolars brackets. The customized lingual bracket bases are often very curved, and they do not permit to measure the adhesive remnant index (ARI) [16], with traditional procedures such as digital photography or scanning electron microscope (SEM) analysis. Confocal scanning microscope presents the advantage of capturing 3D images and found applications in different fields [17, 18] but never in orthodontic for brackets evaluation. The scanning procedure, however, requires a long period of training and the acquisition for every single bracket last between 60 and 90 minutes.

This research aims to perform the first in-vivo analysis of the composite remnants on the bracket surface of a customized lingual appliance.

## Methods

The authors of the present study collected one hundred lingual brackets (25 incisors, 25 canines, 25 premolars, and 25 molars) at the end of orthodontic treatment with customized lingual appliance (Incognito ™, 3M) from 25 patients. Every patient was collected randomly one bracket per type with a general 50/50 ratio between the maxillary and the mandibular dental arches, according to the following inclusion criteria: no antecedent orthodontic treatment, no history of dental trauma, uninjured lingual surface, and no bleaching in the previous 12 months. Patients presenting a Bolton index discrepancy were excluded as well as patients that required an extraction treatment, orthognathic surgery or had impacted teeth. Furthermore, we excluded treatments that lasted more than 24 months. Regarding the molars and premolars, brackets presenting an excessive curvature and 90° undercuts were excluded from the sample because they will have affected the scanning procedure. Sample size calculation revealed that a minimum of 80 brackets (20 per group) were necessary to identify, with a power of 0.80 and a type I error of 5%, a significant difference in resin remnants percentage between the four groups.

The bonding procedures followed the indirect bonding protocol with the clear silicon tray, provided by the manufacturer instruction, and it involved sandblasting the teeth surface (aluminum oxide 50 micron), etching for 30 seconds with orthophosphoric acid 37%, universal bonding and Relyx Unicem. The first debonding phase consisted in removing the appliance with an appropriate lingual debonding plier. Secondly, the operator carefully cleaned up the teeth with a debonding carbide burs at slow speed and performed a final step of enamel polishing with finishing points. The same operator performed all bonding and debonding procedures.

**Fig. 1:**
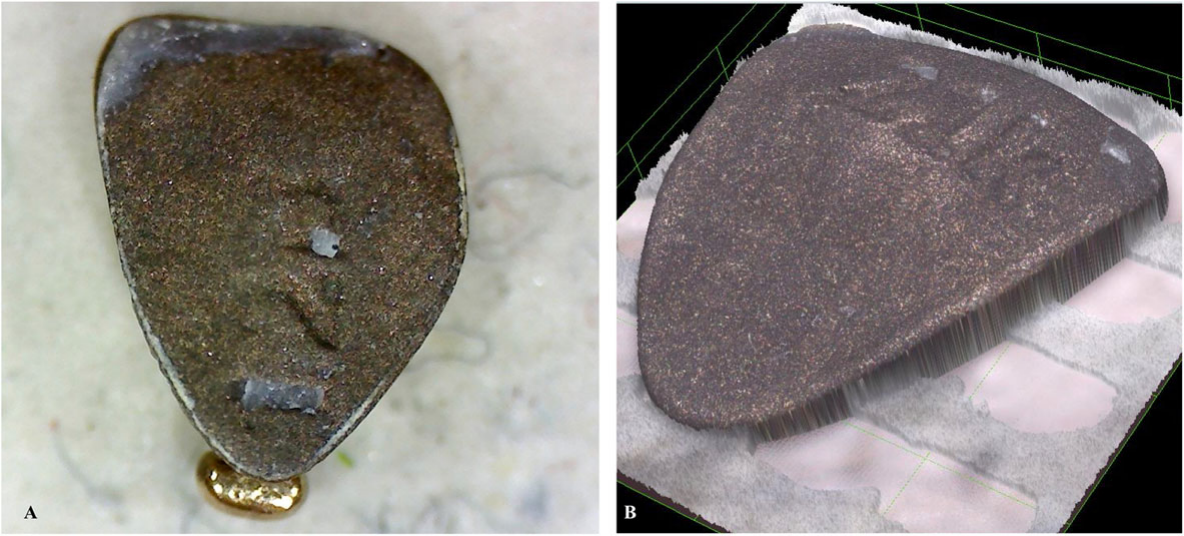
**a** Bracket evaluation at the optical microscope 20x; **b** Three-dimensional scanning with the confocal microscope

Considering the curved surface of lingual brackets, an optical microscope with 20x magnification would have obtained pictures out of focus in some part of the bracket affecting a correct ARI calculation (Fig. 1). This kind of microscope is sufficiently precise for standard vestibular or lingual brackets without a customized base. For this reason, we decided to use a confocal laser microscope (Olympus Lext OLS4000). The confocal laser microscope finds its application for nanometric scale imaging (magnification ranges from 100x to 17000x) and roughness measurements. The high-resolution 3D images of the brackets let us calculate the total base and resin remnants surfaces (Fig. 1).

One operator, skilled in confocal laser microscope imaging, acquired all the 3D images. When the total surface of the bracket exceeded the maximum acquiring area the integrated software automatically merges two or more acquisitions. After the scanning phase, the brackets’ surface and the area of resin remnants were measured with the software of the microscope by a first operator. A second round of measurements was performed by the same operator and by a second operator on all the brackets to calculate the intra-observer and inter-observer agreement on the ARI.

**Fig. 2:**
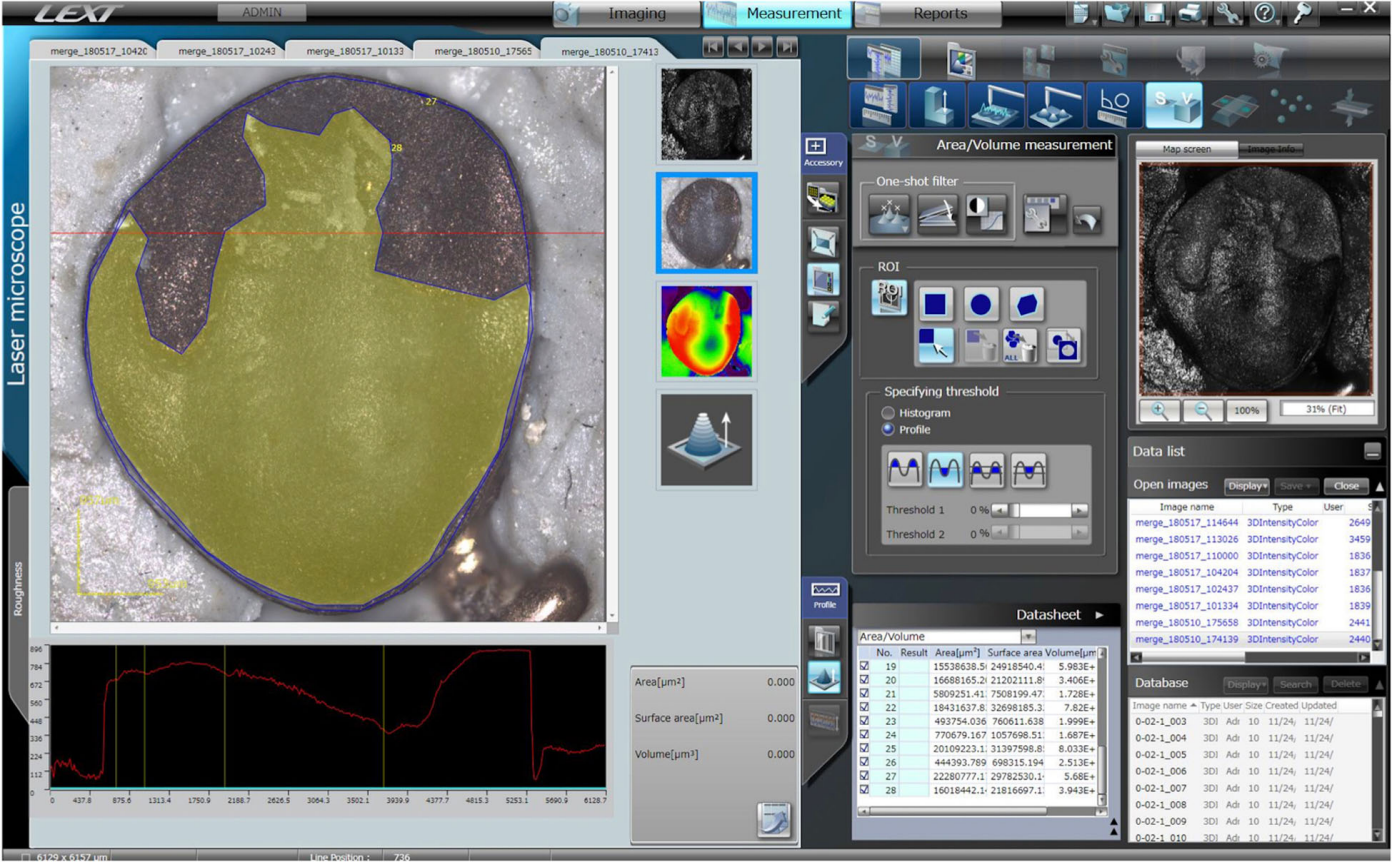
Brackets’ surface and the area of resin remnants were measured with the software of the microscope

The values were calculated as the percentage between the total area and the resin surface as an index of the quantity of adhesive on enamel after debonding (Fig. 2).

ARI calculation was indirect for each tooth, measuring the resin remnants to the total surface of the bracket. ARI’s categories are considered in according with Sfondrini et al. [19]

- ARI 0 no resin remnants on the bracket surface
- ARI 1 less than a half of the bracket covered by resin remnants
- ARI 2 more than a half of the bracket covered by resin remnants
- ARI 3 all the bracket covered by resin remnants.

**Fig. 3:**
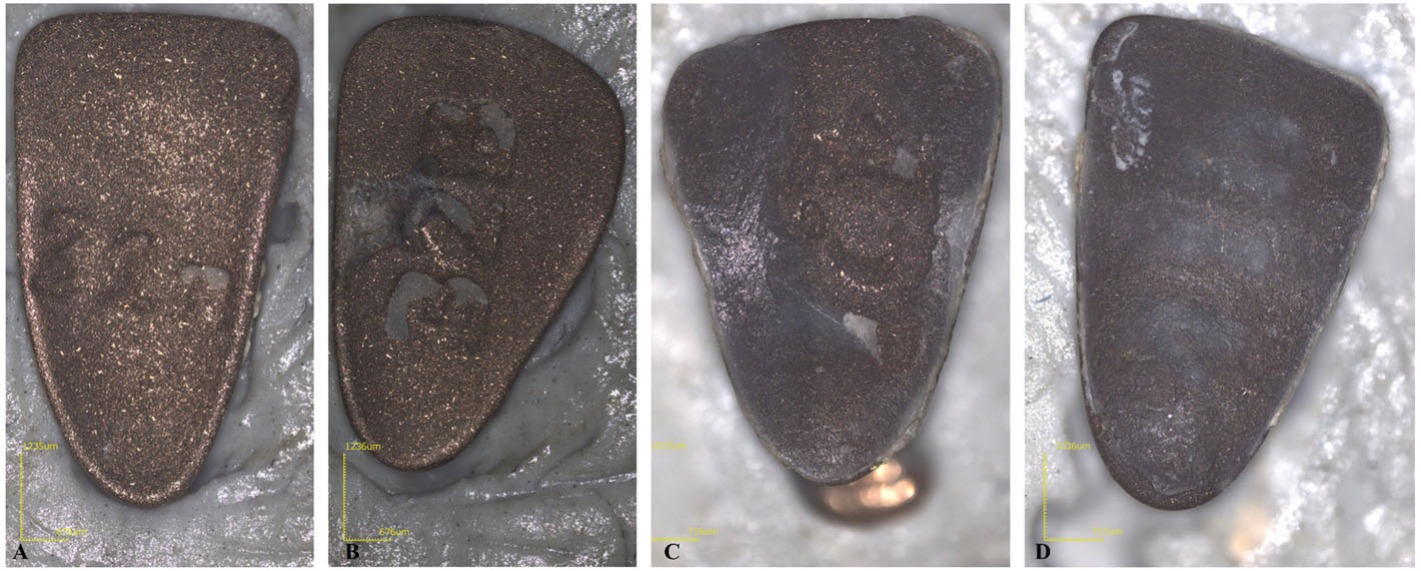
Incisors: **a** ARI 3; **b** ARI 2 **c** ARI 1 **d** ARI 0

**Fig. 4:**
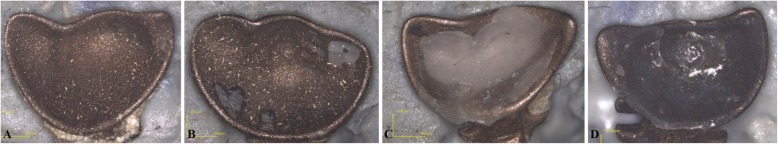
Premolars: **a** ARI 3; **b** ARI 2 **c** ARI 1 **d** ARI 0

Furthermore, the percentage was calculated for each bracket group (Figs. 3 and 4).

### Statistical analysis

Concerning the ARI classification, the inter-observer agreement was assessed with the Fleiss' Kappa and the intra-observer agreement with the Cohen's Kappa. The Shapiro-Wilk test was used to test the normality of the distribution of the primary outcome (resin remnants percentage) evidencing a significance deviation from normality. Median and quartiles were presented to describe the data. The Kruskal-Wallis test and the Fisher test were applied respectively to compare the percentages of remnants and the frequencies of the ARI between the four groups. Differences were considered significant when they reached a p-value < 0.05. The data analysis was carried out with the aid of R 3.5 (R Foundation for Statistical Computing, Vienna, Austria).

## Results

The sample includes 100 brackets, divided into 25 incisors (13 upper and 12 lower), 25 canines (12 upper and 13 lower), 25 premolars (13 upper and 12 lower), and 25 molars (12 upper and 13 lower). Mean bracket surface in the four groups is reported in Table 1.

**Table 1:** Resin remnants as percentage for the different teeth

Teeth	Incisors (*n* = 25)		Canines (*n* = 25)		Premolars (*n* = 25)		Molars (*n* = 25)	
Mean bracket surface (mm^2^)	30.20		30.31		29.30		79.18	
**Min**	0		0		0		0	
**Q1**	2.3		4.3		2.6		19.0	
**Median**	**47.7**		**11.0**		**37.2**		**61.0**	
**Q3**	71.5		46.9		80.7		71.5	
**Max**	100		100		100		100	
Kruskal-Wallis test *p*-value (0.27399)								
	**N**	**%**	**N**	**%**	**N**	**%**	**N**	**%**
ARI								
0 (0%)	4	15	1	4	4	16	4	16
1 (0-50%)	8	31	5	20	7	28	9	36
2 (50-100%)	12	50	18	72	9	36	11	44
3 (100%)	1	4	1	4	5	20	1	4
Fisher test p-value (0.1679)								

To record and measure the amount of adhesive remaining on tooth and bracket surfaces, the adhesive remnant index (ARI) score was used. This scale allows the allocation of a score (0, 1, 2, or 3) depending on the amount of adhesive detected on the enamel surface.

Excellent inter-observer (two observers: Fleiss’ Kappa 0.93) and intra-observer (two classifications of ARI index after 30 days: Cohen’s Kappa 0.97) matches were observed.

**Fig. 5.**
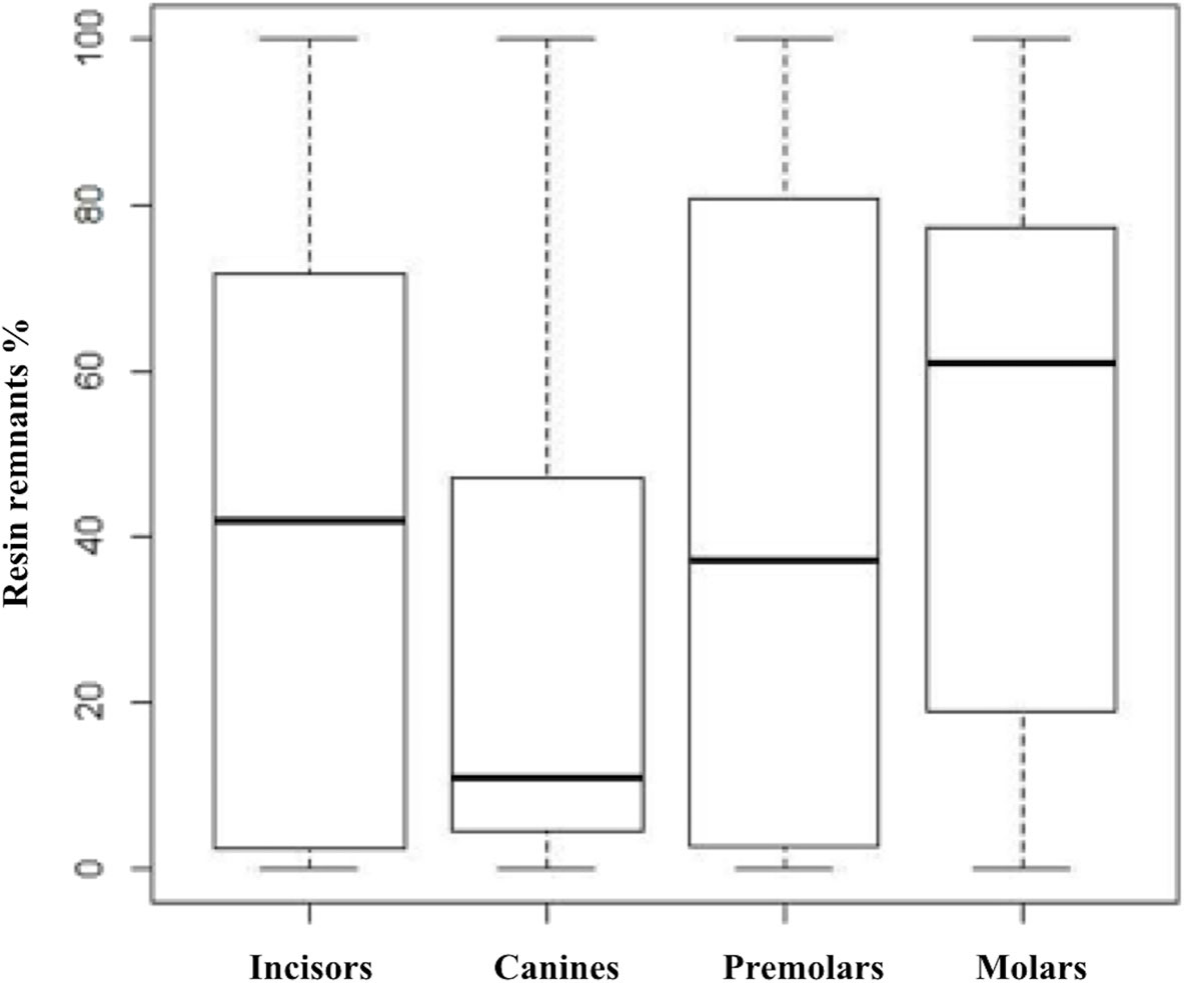
Resin remnants on the brackets (percentage): boxplot

**Fig. 6.**
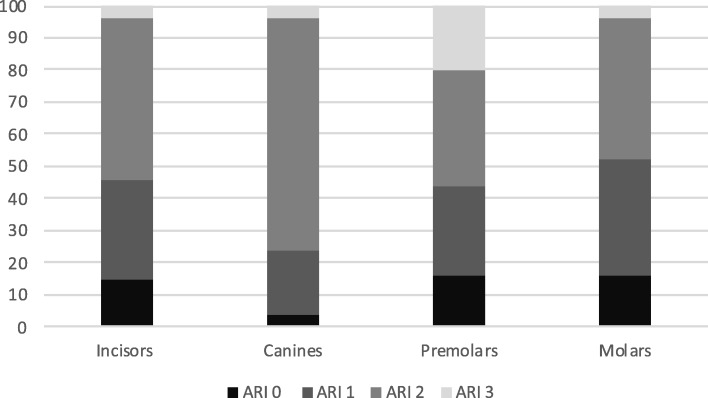
Resin remnants (ARI): histogram

After all the brackets evaluation, the incisors presented 4 brackets with ARI 0, 8 were ARI 1, 12 were ARI 2 and 1 bracket was ARI 3. The canines presented 1 bracket with ARI 0, 5 brackets were ARI 1, 18 were ARI 2 and 1 was ARI 3. The premolars presented 4 brackets with ARI 0, 7 were ARI 1, 9 were ARI 2 and 5 were ARI 3. Finally, molars presented 4 brackets with ARI 0, 9 were ARI 1, 11 were ARI 2 and 1 were ARI 3. Table 1 and in Figs. 5 and 6 extensively report the results in terms of median percentage, quartiles, and ARI distribution in the four groups.

Concerning the whole sample, 13 brackets resulted with no resin surface meaning no adhesive on the bracket, 29 brackets resulted in less than half of adhesive on the surface, 50 brackets resulted in more than half of adhesive on the surface and 8 brackets resulted in all the surface covered of adhesive. Canines are the brackets with the lower remnants of resin followed by premolars, incisors, and molars. Concerning the percentage of resin remnants, Kruskal-Wallis test did not result statistically significant (*p* = 0.27399). No significant differences emerged in the ARI distribution between the four groups, evaluated with the Fisher test (*p* = 0.1679).

## Discussion

Lingual orthodontics, among both young and adult patients, increased in popularity during the last years. Recent studies demonstrated that lingual orthodontics could provide treatment outcomes similar to those achieved with labial appliances.

The orthodontic literature shows many articles that describe the failure of the brackets bonding, the major part of which were made in vitro and on small teeth sample size, usually premolars [2, 5, 6, 11, 13, 20]. There are different limitations of in-vitro studies: the increasing load applied is not representative of the clinical debonding situation that presents a combination of cut, traction, and torsion forces. Furthermore, oral conditions during an orthodontic treatment such as temperature, humidity, salivary pH, and plaque surely influence the debonding result and cannot be simulated in a laboratory.

There are very few studies on the failure of the customized lingual bracket bonding [21–23], for the difficulty of evaluating a curve surface. There are no studies that evaluate it on premolars and molars after orthodontic treatment.

The debonding procedure should preserve the enamel integrity. Resin remnants left on the teeth increase the risk of damaging the enamel during the cleanup procedure with burs. On the other side, its presence on the surface of the brackets is not an advantageous event for the high probability of enamel damage. The frequent prevalence of ARI 2 suggests that the debonding of lingual brackets is comparable with conventional vestibular bracket. Molar customized brackets presented an increased but not statistically significant presence of resin remnant, probably due to the larger bracket surface and their increased convexity. For this reason, the authors of this study suggest paying attention during the debonding procedure of molar brackets since a stronger connection between the adhesive and the bracket mesh means a higher risk of enamel damage [24].

No previous studies evaluated the bracket's base with a confocal laser microscope. The authors captured high-resolution 3D images to measure the entire base and resin remnants in a curved surface and considered this method consistent, precise, and reproducible. On the other side, the confocal laser microscope presented some limits with the brackets with a curvature higher than 85°. Some customized lingual premolars and molars show occlusal extensions with excessive curvature, and for this reason, these brackets were excluded in this study. This study also presents some limitations. First of all, the acquisition is an expensive procedure in cost and time and surely requires a skilled operator. Secondly, the authors did not consider in the study design the percentage of material that can be lost during the debonding procedure.

## Conclusion

No previous studies evaluated ARI on customized lingual brackets after an orthodontic treatment. The method employed included a confocal laser microscope to capture the curved brackets' bases. The principal limitation of this study was that we had to exclude brackets with curvature higher than 85°. These are the first comparative data on the percentage of resin remnants on customized lingual brackets.

Lingual brackets showed a high frequency of ARI 2.

Even no differences between the four groups resulted statistically significant, we observed a slight tendency of more resin remnants on molar brackets, due to half-pad configuration. The debonding procedure requires significant attention to not damage the dental surface.

## Data Availability

The data used to support the findings of this study are available from the corresponding author upon request.

## Abbreviations

ARI: Adhesive remnant index
SEM: scanning electron microscope
